# An evaluation of public, private, and mobile health clinic usage for children under age 5 in Aceh after the tsunami: implications for future disasters

**DOI:** 10.1080/21642850.2014.896744

**Published:** 2014-03-27

**Authors:** Bahie Mary Rassekh, Winnie Shu, Mathuram Santosham, Gilbert Burnham, Shannon Doocy

**Affiliations:** ^a^ johns Hopkins University, Department of International Health, Baltimore, MD, USA; ^b^ University of California at Los Angeles, Department of Social Sciences, USA

**Keywords:** Health systems, Post-disaster relief, Utilization of health services, tsunami, Aceh, Indonesia, Child health, Infant health, Private care, Public care, Internally displaced persons, health policy, rehabilitation, infants and toddlers, children and adolescents, immigrants or migrants

## Abstract

*Background*: Aceh, Indonesia, was the hardest-hit area in the 26 December 2004 Indian Ocean earthquake and tsunami, with more than 500,000 people displaced, 120,000 people dead, and total damages and losses estimated at $4.5 billion. The relief effort following the tsunami was also immense. *Objectives*: This study aimed to determine and assess utilization patterns of formal public versus private and mobile health services for children under age 5 with diarrhea, cough and difficulty breathing, fever, or skin disease and to identify determinants of care usage. *Methods*: A household survey of 962 households was administered to caretakers of children aged 1–5 years. A sample of clusters within Banda Aceh and Aceh Besar were selected and those caretakers within the cluster who fit the inclusion criteria were interviewed. *Results*: Of those caretakers who utilized formal health services as the first line of care for their sick child, 62% used a public health facility, 30% used a private health facility, and 8% used a mobile clinic. In terms of significant factors associated with public, private, and mobile care utilization, mobile clinics were at one side of the spectrum and private clinics were at the other side overall, with public care somewhere in between. This was true for several variables. Mobile clinic users reported the lowest cost of services and medicine and the highest perceived level of accessibility, and private care users reported the highest perceived level of satisfaction. *Conclusions*: Utilization of formal health services for children was quite high after the tsunami. The caretaker's perceived satisfaction with public health services could have been improved. Mobile clinics were an accessible source of health care and could be used in future disaster relief efforts to target those populations that seek less care for their sick children, including displaced populations, and those children whose parents have died.

True learning is that which is conducive to the well-being of the world … . (Bahá’í Writings)

## Introduction

The Indian Ocean earthquake and massive tsunami on 26 December 2004 caused enormous amounts of destruction and affected hundreds of thousands of people. Aceh, on the northern coast of Indonesia's Sumatra Island, was the province most devastated from the tsunami. There more than 15% of the total population was directly affected from the tsunami, with over 500,000 individuals displaced, and nearly 200,000 reported dead or missing. Approximately one-fifth, or 53, of the 244 health facilities in the public sector in Aceh were severely incapacitated or destroyed (WHO, 2005) and one-tenth, or 42, of the province's 481 health professionals died (WHO, 2005) and many others did not work after the tsunami. Total damages and losses to Aceh from the tsunami were estimated at $4.45 billion, which is almost equal to the province's total GDP (BAPPENAS, 2005).

The Indian Ocean tsunami brought with it enormous devastation, and a substantial global relief effort, with several billions of dollars pledged from organizations and governments across the globe. These events caused great changes in the lives of the Acehnese, especially those populations who were displaced from their homes and patterns of life.

### Understanding care-seeking behaviors

Much knowledge can be gained from understanding the practices of people affected by the tsunami in seeking health care for their children, applying some of these findings to other natural disasters.

A successful health strategy depends only in part on the quality of a healthcare system. An equally important factor is the household's behavioral patterns, including care-seeking practices by the primary caregivers (Claeson & Waldman, 2000). Research strategies should increasingly focus on better understanding care-seeking practices (Claeson & Waldman, 2000). Many articles discuss the importance of understanding care-seeking practices in order to meet the health needs of disadvantaged populations (Aggarwal, Kumar, & Kumar, 2003; Barnes-Josiah, Myntti, & Augustin, 1998; Claeson & Waldman, 2000; Darmstadt et al., 2005; Fikree, Ali, Durocher, & Rahbar, 2004; Holts et al., 2003; Mbonye, 2003; Mekonnen & Mekonnen, 2003; Oluwole, Mason, & Costello, 2000; Preker & Carrin, 2004; Reyes et al., 1993; The World Bank, 1995; Walia & Kumar, 1984). Care-seeking behaviors are important to understand because a population's health status depends largely on healthcare usage (Pokhrel & Sauerborn, 2003). Much knowledge can be gained from understanding the practices of internally displaced persons (IDPs) in seeking health care. This paper explores the utilization of formal public, private, and mobile clinics by caretakers of children under age 5.

### Access to care – an important determinant of care-seeking

Access to care is composed of several elements, including distance to care from the home, access to transportation to care, and cost of transportation, care, and drugs. These have been found to be important determinants of care-seeking in many studies (Ahmed, Sobhan, Islam, & Barkat-e-Khuda, 2001; Buor, 2003; Frankenberg, 1995; Habib & Vaughan, 1986; Pandey et al., 2002; Pillai et al., 2003; Pokhrel & Sauerborn, 2003; WHO Regional Committee, 2003) and can likewise present great barriers to reaching care. It was found in a study in Ghana that greater distance to care was highly associated with poorer utilization, and that respondents were willing to travel up to 5 km in order to reach care (Buor, 2003). In that population, amongst those people who lived within 30 minutes of care, 50% regularly used it, as opposed to around 3–4% of people who lived more than 30 minutes away from care. In a paper on access to health care in Indonesia, Frankenberg describes 1 study which found that mortality rates are 40% higher for children who live more than 10 miles from a hospital versus those who live within 3 miles from a hospital, and 54% higher when a doctor was more than 5 miles away as opposed to in the child's village (Frankenberg, 1995).

In this study, access to care was determined at the household level.

### Focus on children under age 5

There is a large and growing gap between rich and poor countries in terms of infant and child mortality (Claeson & Waldman, 2000; World Bank Health-Nutrition-Population, 2002). In poorer countries, children under the age of 5 bear 30% of the burden of disease (The World Bank, 2001; World Bank Health-Nutrition-Population, 2002). Approximately 70% of childhood deaths are due to preventable or treatable causes, such as acute respiratory infections, diarrhea, measles, malaria, and malnutrition (Claeson & Waldman, 2000). Health during childhood is especially important as early illness may lead to further illnesses later in life (Claeson & Waldman, 2000) and the health of a person throughout life is partially dependent on interventions early in one's life, or lack thereof (World Bank Consultation, 2002).

### Public versus private health care

Private health care is increasingly being recognized as an essential part of a country's healthcare system, especially in the developing world. This paper does not include informal providers who are not formally trained or legally recognized in Aceh; all providers mentioned, including public and private providers, are defined as formally trained physicians, nurses, and midwives. Public providers are those practitioners working at a public health facility. Private providers do not work at public health facilities. Private providers can work as private for-profit or can be employed by a private organization, such as non-governmental organizations, private voluntary organizations, or other private organizations. Some practitioners work at both a public facility and a private facility. In this paper, the provider type is defined by the location of the provider at the time of services to the sick child.

In some countries, private-sector providers are more commonly utilized than public-sector providers for childhood illnesses (Ha, Berman, & Larsen, 2002; Peters, Rao, & Fryat, 2003; Waters, Hatt, & Peters, 2003). For example, in Vietnam, the private sector provides 60% of the outpatient care, even though the costs of private care to households are twice as much as for the public sector (Ha et al., 2002). Furthermore, private care is not just used by the wealthier urban populations (Waters et al., 2003).

Often times, even when health care is provided at the public facilities, more money will be spent to receive similar services from a private provider by households, who are often willing to pay for these services which have a higher perceived quality than those provided by a public provider (Waters et al., 2003).

Considering that private care is an important way for children to receive health services in many developing countries, it is important to better understand the role that the private sector played in providing curative health services to sick children after such a large disaster as the tsunami. This paper looks at the utilization of public and private formal health services by caretakers of children under age 5 and considers the specific role that the mobile health clinics played. Given that 78.3% of caretakers sought formal health care as their first line of care for their sick child (Rassekh & Santosham, 2014), this paper analyzes the determinants that are correlated with this formal care usage by the sector.

### Mobile clinics

In order to respond to the emergency situation immediately after the tsunami, there was a large amount of external medical support in the form of mobile clinics. Within two weeks of the tsunami, approximately 130 foreign relief organizations set up mobile clinics and provided staff and medical supplies (Carballo, Daita, & Hernandez, 2005). Many mobile clinics tended to be buses or similar vehicles. One organization, however, that arrived in Aceh on 28 December 2004, utilized helicopter mobile clinics (Médecins Sans Frontières Doctors Without Borders, 2005).

In this paper, it is assumed that the mobile clinics represent a part of the relief effort after the tsunami in Aceh, and studying the utilization patterns and determinants of usage of mobile clinics helped assess this element of the relief effort. It is important to understand the effectiveness of mobile clinics in reaching sick children under age 5, and caretakers' satisfaction with the services, availability of medicine, wait time, and reasons caretakers chose mobile clinics if it was their first line of care chosen.

### Perceived satisfaction

Quality of health care can be defined by its end goal of improving the patient's health (Mahapatra, 2003). However, when patients are interviewed about their perceived satisfaction with health services, there may be several factors that form their level of satisfaction, including their expectations (Newsome, PRHGW, 1999) and interpersonal relationship with the healthcare provider (Rohde & Viswanathan, 1994). Perceived satisfaction can affect a caretaker's choice of where to seek care for their child. Often, if perceived satisfaction is greater in a facility that costs more for services and medicine, caretakers who can afford to go to such a facility will go. If this trend continues, over time it can lead to inequities in the health system, with more affluent members of society utilizing care that is perceived to be of higher quality. Understanding perceived satisfaction with care is therefore very important.

This study was carried out in association with the Johns Hopkins University Center for Refugee and Disaster Response. It was part of the Center's evaluation of the health status and living conditions of IDPs in the Aceh region of Indonesia, affected by the tsunami.

In this study, care-seeking practices of two populations were assessed: IDPs living in barracks and non-IDPs living in their permanent homes. Care-seeking practices of caretakers of children between the ages of 1 and 5 were considered. The conditions for care-seeking that were included were symptoms of the common illnesses that affect children under the age of 5 in this population, including diarrhea, cough and difficulty breathing, skin disease, and fever. The purpose was to determine if non-infant children under the age of 5 utilized the available formal healthcare resources when needed and to explore what determinants were associated with care-seeking formal public, private, and mobile clinics in this population.

## Methods and materials

### Study site

The population included in this study was already self-selected by IDP status. Therefore, a quasi-experimental non-randomized design was utilized to fulfill the objectives of this study.

A cross-sectional study design was used to study the relationship between care-seeking behavior at public and private healthcare services and household IDP status for children under age 5.

The sampling method used was stratified cluster sampling, with sampling units of clusters of population within the Banda Aceh and Aceh Besar areas. Barracks exist in groups and were best divided as clusters of homes. Forty-eight clusters of 24 households each were to be interviewed in this study, since around 30 clusters enable the study estimates to be precise (Robinson, 2003).

Disproportional stratification was used, such that the sample size for each strata was calculated independently of that characteristic's natural occurrence in the population. In this situation it was important to utilize this method of stratified sampling in order to ensure that a detectable difference could be seen in the primary outcome measures.

In this study, inclusion depended on need for care as determined by the caretaker's recognition of the following conditions: diarrhea, difficulty breathing and cough, skin disease, or fever. In this study, these symptoms were determined by the caretaker.

### Data collection

The main data collection instrument was a survey conducted by trained interviewers who were enrolled or recently graduated university students in Banda Aceh. There were four teams of six interviewers and one supervisor each. The survey was administered in the local language, Bahasa Indonesia, 9–10 months post-tsunami. The interviewers and supervisors were trained prior to the commencement of data collection and a field manual was given to each person to better understand the process and to refer to throughout the study.

In order to ensure that participants consented to being included in the study, interviewers read an introductory consent statement and asked all respondents to sign it before proceeding with the survey. In the case that the respondent was unable to sign it, verbal consent was given and the interviewer took note. In order to maintain confidentiality, participants' full names were not recorded on the survey. At the end of the interview, interviewers reviewed completed questionnaires in order to ensure that all questions were filled out completely and appropriately.

The survey was translated into Bahasa Indonesia from English, and then back-translated in order to ensure accuracy and maintain the integrity of the questions and their intended meaning.

The survey was pilot-tested for approximately 30 individuals before official data collection commenced. This provided an opportunity to field-test the survey instrument and to further refine the questions in order to ensure accuracy and to enable evaluation of question precision in Bahasa Indonesia. Some questions were open-ended during the pilot test in order to allow caretakers to answer them as they saw fit. The data gathered from these questions were categorized and common responses were added to the questionnaire for inclusion in the official data collection process. This also allowed an opportunity for project investigators to gain a better understanding of local customs and common practices used for treating childhood illness. This information enriched and strengthened the survey instrument and allowed for a more fruitful research process.

Although great care was taken to get accurate lists of all the barracks from various government offices, none of the lists accurately reflected all functioning barracks. Several barracks were listed that were not being used, mostly because people had not yet moved into them. This affected the study's household selection process. Originally, using the most complete list possible from the government office in Banda Aceh, barracks were randomly selected to be included in the study. Once teams went into the field to collect data, if a barrack was not in operation, then another barrack in the same area was chosen to be visited. If that barrack also was not operational, then the next barrack would be chosen, until one was found. Clustering in terms of non-IDP neighborhoods remained as was intended. Overall, there were 28 clusters with 962 observations.

Data were entered, and then re-entered, in Microsoft Access by trained data-entry people locally hired in Indonesia. Double entry was conducted by a different person entering the same data for a second time. Discrepancies between the two sets of entered data were then checked against the original form. Double entry and discrepancy checking ensured accuracy and completeness of the data, so that it reflected the raw data. Once data were entered, they were analyzed.

The survey included questions regarding utilization of health services and characteristics of the caretaker, household, and child. These included the caretaker's age, education level, gender, household income source and savings amount, number of children for which the caretaker is caring, distance to the health facility, age and gender of the child, and cost of services, among others.

The survey also included questions regarding perceived satisfaction with the health services received. Eight questions were asked. The first question asked regarding their level of satisfaction in general. The following seven questions asked about the respondents' level of satisfaction with the health provider, the time spent to obtain services, interaction with the facility staff, and equipment and supplies. Each question was measured using a five-point Likert scale (i.e. extremely satisfied, satisfied, neutral, dissatisfied, and extremely dissatisfied). Principal components analysis was used to determine that only one factor was necessary to capture the contribution of all eight questions. Using a factor analysis, each respondent was given one overall score of perceived satisfaction with the health services received. The score was based on each question's answer, which was first standardized, and then multiplied by the question's assigned loading. For each person, all eight question values were added to come up with that person's final score.

### Setting and participants

The study was conducted in urban Banda Aceh, in the province of Aceh, Indonesia. This province was closest to the epicenter of the earthquake, was hit the hardest by the tsunami, and was home to over 500,000 displaced tsunami victims.

### Comparison groups and selection criteria (Figure 1)

The sample size was calculated to allow for a power of 80% (1* *–* β *= 0.80).

**Figure 1.**
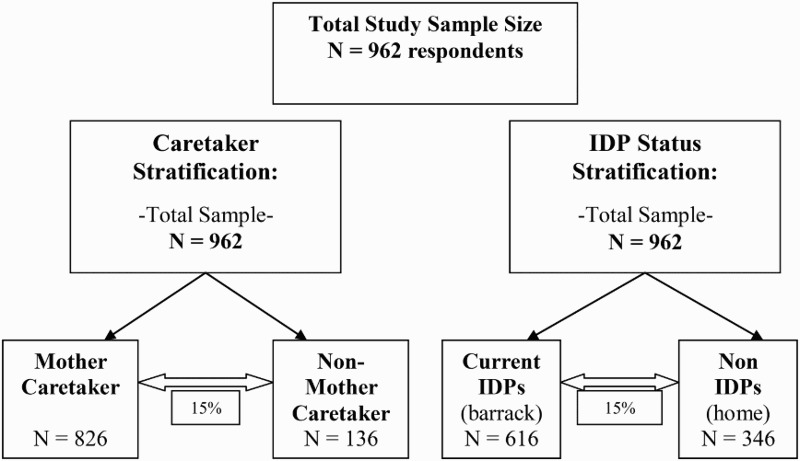
Survey sampling frame.

### IDP status comparison groups

#### IDP households

*Inclusion criteria:* Internally displaced people who lived in a barrack community at the time of the survey, and who had lived in a barrack community for at least two weeks, were included in this category. Within the household there had to be a child between the ages of 1 and 5 who had a fever, cough and difficulty breathing, diarrhea, or skin disease in the two weeks prior to the survey.

#### Non-IDP households

*Inclusion criteria:* People who lived in a home at the time of the survey, and who had lived in that home since before the tsunami, were included in this category. Within the household there had to be a child under the age of 5 who had a fever, cough and difficulty breathing, diarrhea, or skin disease in the two weeks prior to the survey.

### Type of caretaker comparison groups

#### Caretaker type: mother of child

*Inclusion criteria:* A child who was cared for at the time of the interview by her/his mother was included in this category. That child had to be between the ages of 1 and 5 and have had a fever, cough and difficulty breathing, diarrhea, or skin disease in the two weeks prior to the survey.

#### Caretaker type: non-mother of child

*Inclusion criteria:* A child who was cared for at the time of the survey by anybody but her/his mother was included in this category. This included the child's father, other family member, or non-family member. That child had to be between ages 1 and 5 and have had a fever, cough and difficulty breathing, diarrhea, or skin disease in the two weeks prior to the survey.

## Results – public versus private care usage

A brief summary of some of the results obtained from this research is as follows. There was observed to be a significant difference between the cost of medical services for private, public, and mobile clinics. The amount spent on private care was about eight times the amount spent on public care. Likewise, there was seen to be a 10-fold difference in the price of medicine in public versus private care. In terms of households returning to health care: of 955 households, 320 (33.5%) returned because of the good cost of health care, 344 (36.0%) returned because care was easy to access, 280 (29.3%) returned because of the perceived good quality of the care or provider, and 11 (1.2%) returned because their family supported this care option. It was shown that caretakers who had more education had a slightly greater odds of utilizing public health care. Finally, it was found that the gender of the child was a significant variable in the utilization of mobile clinics versus public care.

### Subjects

There were 962 caretakers of children from 962 different households that were interviewed in this study. Of these caretakers, 918 were included in the multiple logistic regression (MLR) analysis, representing 918 unique households.

Of the 962 caretakers interviewed, 646 (67.2%) utilized public primary care. In Aceh, these are called “puskesmas”. Three hundred and sixteen (32.8%) utilized private clinics as their first line of health care for their sick child.

#### Statistically significant factors

##### Reason chose care option

**Figure 2.**
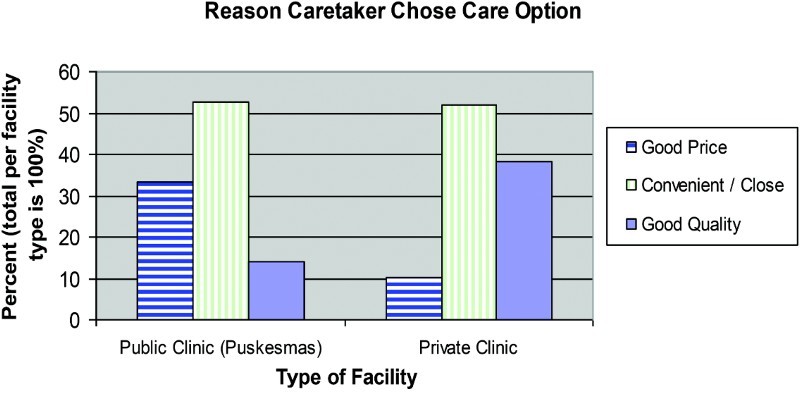
Reason caretaker chose public or private facility for child's illness.

An MLR analysis was conducted, adjusting for variables of the child's age and gender, the child's disease, if the child was the eldest, age and education of the caretaker, distance to a formal healthcare facility, main reasons to return to health options again, living status of the child's parents, household savings, whether the caretaker's mood is still affected by the tsunami, and other variables related to care usage (Table 1). Relative to those who chose their healthcare option because of the good price, those who chose it because it was convenient or close, had 2.3 times the probability of seeking private versus public care (*p* = .001). Those caretakers who chose it because of perceived good quality had an odds of 2.8 of utilizing private versus public care (*p* = .008) (Figure 2).

##### Wait time to see healthcare provider

Wait time to see a healthcare provider was found to be an important variable in the utilization of private health services by caretakers of children under age 5. On average, respondents waited 14.0 minutes before seeing the healthcare provider, with 14.7 minutes on average at puskesmas and 12.6 minutes on average at private clinics.

Using the adjusted MLR, the likelihood of utilizing a private clinic versus public health care decreased by 7.3% for every one minute increase in time the caretaker had to wait to be seen, for those who waited 15 minutes or less (*p* < .001). For wait time that was above 15 minutes, the probability of utilizing a private versus a public clinic was 1.1 for each additional minute of wait time (*p* < .001).

**Table 1.** Crude and adjusted odds ratios of utilization of formal public versus private health clinics.

	Crude (*n *= 962)			Adjusted (*n *= 918)		
	Odds ratio	95% CI	*p*-Value	Odds ratio	95% CI	*p*-Value
IDP status	1.209	(0.915, 1.597)	.182	0.753	(0.445, 1.273)	.289
Age of child (years)	0.916	(0.807, 1.040)	.175	0.924	(0.803, 1.062)	.266
Gender of child (female versus male)	0.963	(0.736, 1.261)	.786	1.086	(0.787, 1.500)	.614
Child's illness						
Reference* *= No diarrhea and no cough and difficulty breathing (cough/difficulty breathing)						
Diarrhea but no cough/difficulty breathing	0.640	(0.367, 1.114)	.115	0.569	(0.311, 1.039)	.066
Cough/difficulty breathing but no diarrhea	0.924	(0.685, 1.249)	.788	1.081	(0.652, 1.794)	.762
Diarrhea and cough/difficulty breathing	0.917	(0.577, 1.457)	.715	1.298	(0.825, 2.042)	.259
Distance to health facility (meters)	1.000	(1.000, 1.000)	.070	1.000	(1.000, 1.000)	.401
Reason chose care option						
Reference* *= Good price						
Convenient/close	3.277	(2.151, 4.993)	.000	2.305	(1.384, 3.837)	.001
Good quality	8.951	(5.620, 14.257)	.000	2.848	(1.317, 6.161)	.008
Time waited to see care provider (minutes, 0–15 minutes)	0.992	(0.983, 1.000)	.073	0.926	(0.909, 0.943)	.000
Spline term (16 or more minutes)	1.010	(0.998, 1.023)	.097	1.120	(1.087, 1.156)	.000
Cost of services (rupiah)	1.101	(1.072, 1.131)	.000	1.086	(1.051, 1.121)	.000
Willingness to pay for services						
Reference: Willing to pay 0–6000 rupiah						
Willing to pay maximum amount (12,000 rupiah)	3.947	(2.958, 5.266)	.000	2.902	(1.649, 5.108)	.000
Medicine available at facility (no versus yes)	1.435	(0.884, 2.331)	.884	2.165	(0.991, 4.730)	.053
Delay in care-seeking (days after saw symptoms)	1.129	(0.814, 1.568)	.467	1.190	(0.929, 1.525)	.168
Main reason return to health option again						
Reference* *= Good cost						
Easy to access	2.593	(1.770, 3.800)	.000	1.587	(0.852, 2.957)	.145
Good quality care/provider	6.354	(4.318, 9.350)	.000	3.554	(2.190, 5.770)	.000
Family supports it	9.917	(2.795, 35.178)	.000	2.861	(0.917, 8.932)	.070
Child first/eldest child (yes versus no)	1.028	(0.785, 1.346)	.842	1.095	(0.715, 1.676)	.677
Mother and father of child living						
Reference* *= Both mother and father living						
Only mother living	0.389	(0.180, 0.842)	.017	0.435	(0.186, 1.016)	.054
Only father living	0.834	(0.377, 1.843)	.654	0.945	(0.373, 2.396)	.905
Neither living	0.834	(0.214, 3.248)	.793	0.549	(0.136, 2.218)	.400
Age of caretaker (years, 12–30 years)	1.010	(0.992, 1.029)	.272	0.982	(0.906, 1.063)	.651
Spline term (over 30 years)	1.018	(0.995, 1.042)	.227	1.052	(0.974, 1.136)	.199
Education of caretaker (years school attended) (>12 years versus <12 years)	1.224	(0.935, 1.604)	.141	0.710	(0.518, 0.973)	.033
Main source of health information						
Reference* *= Posyandu						
Television, radio, newspaper	1.016	(0.665, 1.553)	.942	1.251	(0.662, 2.364)	.491
Health Team	1.138	(0.834, 1.551)	.415	1.284	(0.780, 2.114)	.326
Other source (neighbors, friends, relatives, private provider, school, no source, etc.	1.194	(0.658, 2.164)	.560	1.556	(0.577, 4.915)	.383
Tsunami affects mood now (no versus yes)	1.282	(0.956, 1.718)	.097	1.206	(0.774, 1.878)	.408
Current source spending money/income						
Reference: Earning money, and no government support or non-family support						
Government support or non-family support, and not earning money	0.660	(0.353, 1.234)	.193	1.079	(0.375, 3.101)	.888
Earning money and government or non-family support	0.800	(0.527, 1.214)	.294	0.617	(0.407, 0.936)	.023
Neither earning money nor receiving government or non-family support	0.840	(0.394, 1.793)	.653	0.934	(0.430, 2.032)	.864

##### Cost of services

Cost of services was also found to be an important factor associated with utilizing private health services by caretakers of children under age 5. On average, respondents spent 2803 rupiah (USD 0.28) for this particular visit to the health service for their sick child, with an average of 851 rupiah (USD 0.09) at the puskesmas and 6796 rupiah (USD 0.68) at the private clinics. The amount spent on private care was about eight times the amount spent on public care. This is a significant difference.

The amount spent for medicine when it was available was also significantly different at the public and private care facilities. On average, the cost of medicine was 1631 rupiah at the public facility and 16,690 at the private facility, more than a 10-fold proportion.

In the MLR, the adjusted odds or probability of utilizing private versus public health care had a ratio of 1.1 for each 1000 rupiah (approximately $0.10) increase in cost of services (*p* < .001).

##### Willingness to pay for services

A caretaker's willingness to pay for the same health services as the one they received was also found to be an important variable that was associated with utilizing private health services. Of the 962 households interviewed in this study, 944 (98.1%) responded to this question. Of these 944 households, 588 (62.3%) said that they were willing to pay between 0 and 6000 rupiah for the same services received; 356 (37.7%) were willing to pay the maximum amount that was asked in the survey which was 12,000 rupiah.

In the MLR, the probability of seeking private versus public care among those who were willing to pay the maximum amount of 12,000 rupiah for services was 2.9 times that of those who were not willing to pay anything or up to 6000 rupiah (*p *< .001).

##### Reason to return to healthcare option

The reason that caretakers return to healthcare options was also found to be an important factor associated with utilizing private health services for their under-five children. Of the 962 households interviewed in this study, 955 (99.3%) responded to this question. Of these 955 households, 320 (33.5%) returned because of the good cost of health care; 344 (36.0%) returned because care was easy to access; 280 (29.3%) returned because of the perceived good quality of the care or provider; and 11 (1.2%) returned because their family supported this care option.

In the MLR, comparing each of the three latter categories of reason to return to health care to the category of returning because of good cost, the reason of “good quality care and provider” was significantly different. The likelihood of using private care among those caretakers who used this response was 3.6 times of those who stated “good cost” (*p *< .001).

##### Caretaker's education level

The level of the caretaker's education was found to be an important factor associated with utilizing public health services. Of the 962 caretakers interviewed in this study, 962 (100%) responded to this question. Of these respondents, 471 (49.0%) had 0–11 years of schooling and 491 (51.0%) had 12 or more years of schooling. In the MLR, those who had 12 or more years of schooling compared to those with 11 or less years of schooling had a 0.7 odds of seeking private versus public care (*p *= .033). Therefore, those caretakers who had more education had a slightly greater odds of utilizing public health care.

##### Current source of household spending money

For those who were earning income and receiving government or other non-family financial support, compared to those who were earning income but not receiving any other financial support, there was a lower probability of utilizing private care (odds ratio: 0.6; *p *= .023).

#### Borderline significant factors

The following variables were not statistically significant at an alpha of 0.05 level in the MLR. However, their *p*-values were less than .100 and represent interesting trends that could be studied further.

##### Illness of the child

The trend in utilization of public and private health care depending on the child's illness was that compared to those children who had neither diarrhea nor cough and difficulty breathing, those who only had diarrhea had a 0.569 odds of seeking private versus public care (*p *= .066). Those children who had only cough and difficulty breathing or had both diarrhea and cough and difficulty breathing sought slightly more private care although the difference was not statistically significant.

##### Medicine availability

Medicine availability at the health facility used for the child in this illness episode was also found to be an important factor associated with utilizing private health services. Of the 962 households 888 (92.3%) said that medicine was available at the health facility. Seventy-four respondents (7.7%) said that it was not available.

In the MLR, those who said medicine was available had a 2.2 odds of utilizing private versus public care (*p *= .053).

##### Only mother alive

Compared to those for whom both the father and the mother of the child were alive, those for whom only the mother was alive had a 0.4 odds of seeking private versus public health care for the sick child (*p *= .054). Therefore, they tended to use more public than private health care.

#### Non-significant factors

Several variables in the MLR were not significantly associated with utilizing private or public health care. These variables included IDP status, age of the child, gender of the child, distance to health care, delay in seeking health care, whether the child was the eldest child, caretaker's age, the main source of health information, and the effect of the tsunami on the mood of the caretaker (see results in Table 1).

#### Effect modification

Effect modification was tested for several variables including: reason return to care option by IDP status, parent living status by IDP status, whether the tsunami affects the mood of the caretaker by IDP status, and reason chose care option by price paid for services. None of these were significant in a model that converged.

## Results – public care versus mobile clinic usage

### Subjects

Seven hundred and thirty caretakers of children representing 730 different households were included in this study. Of these caretakers, 700 were included in the MLR analysis, representing 700 unique households.

Of the 730 caretakers interviewed, 646 (88.5%) utilized public primary care. In Aceh, these are called puskesmas. Eighty-four (11.5%) utilized private mobile clinics as their first line of health care for their sick child.

#### Statistically significant factors

*Gender of the child*. The gender of the child was found to be a significant variable in utilization of mobile clinics versus public care by the under-five children's caretakers. Sick girls relative to sick boys were 1.4 times as likely of being taken to a mobile clinic versus a public facility (*p* = .017).

##### Wait time to see healthcare provider

Wait time to see a healthcare provider at the mobile clinic versus a public clinic was found to be an important variable in utilization of mobile clinics by caretakers of children under age 5. On average, respondents waited 14.7 minutes at the puskesmas and 10.9 minutes at the mobile clinics.

Using the adjusted MLR, the odds of utilizing a mobile clinic was 0.9 for every one minute increase in time the caretaker had to wait to be seen, for those who waited 15 minutes or less (*p* < .001). For wait time that was above 15 minutes, the likelihood of utilizing a mobile clinic versus public care was 1.1 for each additional minute of wait time (*p *< .001).

##### Willingness to pay for services

A caretaker's willingness to pay for the same health services as the one they received for their under-five child for this illness episode in a mobile clinic was also found to be an important factor associated with utilizing mobile clinics versus public clinics.

In the MLR, those who were willing to pay up to 1500 rupiah for the same health services as the one they received had the smallest odds of using mobile health clinics. Compared to them, all other people who completed this question (those who were willing to pay 3000, 6000, and 12,000 rupiah or not willing to pay anything) had a greater likelihood of utilizing mobile clinics versus public clinics as their first line of health care for their sick child. The odds ratios ranged from 4.4 to 10.4, with *p*-values ranging from .037 to .180 (Table 2).

**Table 2.** Crude and adjusted odds ratios of utilization of formal public health clinics versus private mobile health clinics.

	Crude (*n *= 730)			Adjusted (*n *= 700)		
	Odds ratio	95% CI	*p*-Value	Odds ratio	95% CI	*p*-Value
IDP status	0.286	(0.149, 0.550)	.000	0.286	(0.068, 1.193)	.086
Age of child (years)	0.877	(0.708, 1.087)	.232	0.871	(0.681, 1.114)	.270
Gender of child (female versus male)	1.224	(0.774, 1.934)	.387	1.431	(1.066, 1.922)	.017
Child's illness						
Reference* *= No diarrhea and no cough and difficulty breathing (cough/difficulty breathing)						
Diarrhea but no cough/difficulty breathing	0.490	(0.166, 1.447)	.197	0.704	(0.255, 1.943)	.498
Cough/difficulty breathing but no diarrhea	0.947	(0.570, 1.572)	.832	1.069	(0.563, 2.027)	.839
Diarrhea and cough/difficulty breathing	1.105	(0.527, 2.317)	.791	1.090	(0.509, 2.330)	.825
Distance to health facility (meters)	1.000	(0.999, 1.000)	.003	1.000	(0.999, 1.000)	.511
Reason chose care option						
Reference* *= Good price						
Convenient/close	3.077	(1.654, 5.727)	.000	1.601	(0.790, 3.245)	.192
Good quality	1.085	(0.400, 2.944)	.872	0.382	(0.120, 1.217)	.104
Time waited to see care provider (minutes, 0–15 minutes)	0.975	(0.953, 0.998)	.030	0.911	(0.864, 0.961)	.001
Spline term (16 or more minutes)	1.009	(0.989, 1.030)	.373	1.149	(1.075, 1.228)	.000
Cost of services (rupiah)	0.955	(0.867, 1.050)	.338	1.001	(0.900, 1.112)	.992
Willingness to pay for services						
Reference: Willing to pay minimum amount (1500 rupiah)						
Willing to pay 3000 rupiah	4.207	(0.909, 19.473)	.066	5.775	(1.087, 30.690)	.040
Willing to pay 6000 rupiah	3.094	(0.619, 15.461)	.169	4.379	(0.507, 37.832)	.180
Willing to pay 12,000 rupiah	4.506	(1.039, 19.544)	.044	10.380	(1.147, 93.932)	.037
Not willing to pay anything	4.026	(0.940, 17.240)	.061	5.167	(0.854, 31.243)	.074
Medicine available at facility (no versus yes)	2.505	(1.287, 4.875)	.007	1.595	(0.652, 3.902)	.306
Delay in care-seeking (days after saw symptoms)	1.083	(0.621, 1.887)	.780	1.367	(0.851, 2.195)	.196
Main reason return to health option again						
Reference* *= Good cost						
Easy to access	2.847	(1.579, 5.136)	.001	1.828	(0.881, 3.795)	.105
Good quality care/provider	2.667	(1.370, 5.191)	.004	3.330	(1.471, 7.541)	.004
Family supports it	12.000	(2.484, 57.975)	.002	22.513	(3.035, 167.026)	.002
Child first/eldest child (yes versus no)	1.230	(0.777, 1.946)	.376	1.784	(0.888, 3.585)	.104
Age of caretaker (years, 12–30 years)	0.979	(0.945, 1.014)	.244	0.954	(0.866, 1.051)	.339
Spline term (over 30 years)	0.974	(0.925, 1.026)	.320	1.029	(0.930, 1.139)	.575
Education of caretaker (years school attended) (>12 years versus <12 years)	0.732	(0.462, 1.160)	.184	0.783	(0.425, 1.442)	.432
Main source of health information						
Reference* *= Posyandu						
Television, radio, newspaper	0.909	(0.402, 2.055)	.818	1.473	(0.400, 5.426)	.561
Health team	1.666	(0.970, 2.860)	.064	1.543	(0.643, 3.700)	.331
Other source (neighbors, friends, relatives, private provider, school, no source, etc.	1.364	(0.484, 3.844)	.557	1.441	(0.313, 6.625)	.639
Tsunami affects mood now (no versus yes)	0.742	(0.428, 1.287)	.288	0.737	(0.298, 1.819)	.508
Current source spending money/income						
Reference: Earning money, and no government support or non-family support						
Government support or non-family support, and not earning money	0.794	(0.274, 2.296)	.670	0.757	(0.081, 7.066)	.807
Earning money and government or non-family support	0.607	(0.269, 1.367)	.228	0.329	(0.094, 1.145)	.081
Neither earning money nor receiving government or non-family support	1.639	(0.602, 4.460)	.334	1.604	(0.512, 5.024)	.418

##### Reason to return to healthcare option

The reason that caretakers return to healthcare options was also found to be an important factor associated with utilizing mobile clinics for their under-five children versus public care. The reasons included good cost, easy to access, good quality care, and family supports it.

**Figure 3.**
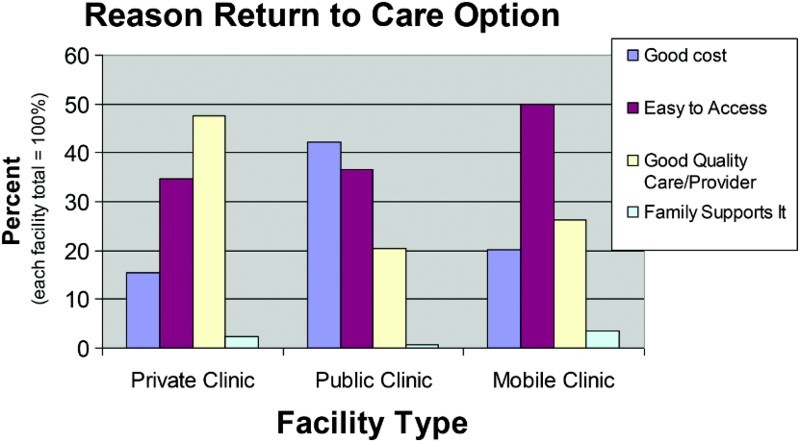
Reason caretakers returned to care options, by type of facility visited for child's illness.

In the MLR in each of the two latter categories of reason to return to health care to the category of returning because of good cost, each comparison produced significant results, and in each comparison there was an increased probability of utilizing private care. The ratio of odds of returning because of good quality care was 3.3 (*p *= .004), and the odds of returning because family supports it were 22.5 (*p *= .002). Comparing the reason to return of easy to access versus good cost, there was no significant difference (Figure 3).

#### Borderline significant factors

The following variables were not statistically significant at an alpha of 0.05 level in the MLR. However, their *p*-values were less than .100 and represent interesting trends that could be further studied.

##### IDP status

Those who were displaced had a greater odds of using mobile clinics than their non-IDP counterparts, adjusting for all other variables in the MLR. The odds of using mobile care for non-IDPs versus IDPs was 0.3 (*p *= .086).

##### Current source of household spending money

For those who were currently earning income and receiving government or other non-family financial support, compared to those who were earning income but not receiving any other financial support, the odds of utilizing mobile clinics versus public care were 0.3 (*p *= .081).

#### Non-significant factors

Several variables in the MLR were not significantly associated with utilizing mobile clinics versus puskesmas. These variables included age of the child, illness of the child, distance to health care, the reason for choosing a particular healthcare option, the cost of services, availability of medicines at the healthcare facility, delay in seeking care, whether the child was the eldest child, the caretaker's age and education, the caretaker's main source of health information, and whether the tsunami still affected the caretaker's mood (see results in Table 2).

#### Effect modification

Effect modification was tested for several variables including: amount willing to pay for services and reason to return to a care option; amount willing to pay for services and reason chose care option; and price paid for services and reason to return to a care option. None of these were significant in a model that converged.

**Figure 4.**
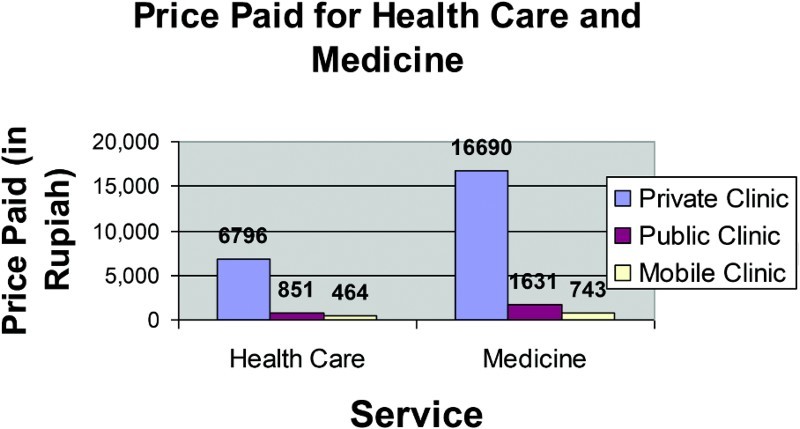
Price paid for health services and medicine for caretakers of children under age 5.

Comparing private facilities, public facilities, and mobile clinics, the price paid for health services and medicine was quite different. Private clinics were the most expensive and mobile clinics were the least expensive (Figure 4).

## Discussion

After the tsunami, many public health facilities and providers did not return to work. Fifty-three of the 244 public health facilities were severely incapacitated or destroyed in the tsunami (WHO, 2005). Forty-two of the province's 481 health professionals died (WHO, 2005) and many others did not work after the tsunami. The public healthcare system, in cooperation with the private care providers, and the relief efforts, managed to provide accessible health care in terms of distance, cost, and medicine availability to both displaced and non-displaced populations residing in the study area. There were differences in reasons for usage of each and perceived strengths of each type of care option. A key recommendation coming from this area of study is the potential for increasing use of mobile clinics. As further explained below, they proved effective and could be used to target specific populations in other post-disaster environments.

Approximately 10 months after the tsunami, 62% of caretakers who used formal care used a public health facility, 30% used a private health facility, and 8% used a mobile clinic. In terms of a spectrum, it could be said that mobile clinics were at one side of the spectrum and private clinics are at the other side overall, with public care somewhere in between. This was true for several variables measured in this study, including the cost of services, cost of medicine, perceived level of accessibility, reasons caretakers chose these care options, and reasons they would return to care options.

Overall, care was reported at about half the cost at the mobile clinics than at the public clinics, including cost of services and medicine, as mobile clinic services were for the most part provided free of charge by the relief community after the tsunami. Respondents who used private care reported greater perceived satisfaction with the services. These trends were demonstrated in several ways.

In the actual reported cost of services, public care was nearly one-eighth the price of private care and mobile clinics were reported at approximately half the cost of public care. The cost of medicine for those who used public care was about one-tenth the cost on average compared to those who used private services and those at mobile clinics reported that the cost was approximately half of public care. In the perception of satisfaction indicator, there was a statistically significant difference between perceived satisfaction for those who utilized public versus private care. Those who utilized private care had greater perceived satisfaction (*p *= .016).

These two trends were also apparent in the reason that caretakers chose their care options. For those caretakers who used public services, more than three times the proportion listed good price as their reason for choosing their care option compared to those caretakers who used private services. That care was convenient or close was the most frequently used reason for choosing a care option for those caretakers who utilized mobile clinics as the first line of care during their child's illness episode. On the contrary, for those caretakers who used private services, nearly three times the proportion listed good quality as their reason for choosing their care option compared to those caretakers who used public services. In a separate question, when asked for the main reason that a caretaker would return to a care option again, those who used public care were just about three times as likely to list good cost as their main reason, compared to those who used private care for their child's illness. Those who used private care were 2.3 times as likely to list good quality as their main reason, compared to those who used public care. When asked for the main reason that a caretaker would return to a care option again the most frequently given answer for those who used mobile clinics listed that they are easy to access.

Given the extremely low cost and perceived convenience of mobile clinics as sources of health care for sick children, this element played an important role of the relief effort in Aceh after the tsunami. However, overall only 11.5% of the caretakers interviewed had used mobile clinics for their sick children. This may be due to the fact that the study was conducted 9–10 months post-tsunami, and that this component of the relief effort could have decreased by that point, although this care option could have continued to be useful, especially for the displaced and the poorest members of the population.

Caretakers had higher perceived satisfaction with private care and utilized it more when quality of care was the reason they chose health services or the reason they would return to them in the future. Private care users were willing to pay more for the same services received than those who used public care. When people wanted to use perceived good quality care they had to spend more money, since private care was more expensive. This may not always be accessible to all populations, and especially to those who are burdened financially, as so many were after the tsunami. Therefore, if people have to spend more money to get greater perceived quality of health care for their children, in the future, those with higher income may utilize more private care and those with lower income may utilize more public care.

The public health system provided low-cost accessible care to the population in Aceh after the tsunami, and about two-thirds of respondents utilized this care option for their sick child during the illness episode focused on in the survey. After a disaster like the tsunami it was important that public facilities were available at low cost to both displaced and non-displaced tsunami survivors. This was especially meaningful given the poverty of the population and loss of life, jobs, and assets that came with the tsunami. Accessible low-cost formal health care helped ensure the financial welfare of the survivors, enabling caretakers to seek care for their children without being unnecessarily burdening financially.

Although public care was very accessible and at low cost 10 months after the tsunami, in the future one area that could be improved in the public healthcare system is the perceived quality of care and perceived satisfaction that caretakers had with the health services they received. This difference in perceived quality and satisfaction could be due to an actual difference or to pre-conceived notions of quality of private and public services. More research could be done regarding this difference in this setting or one similar to it. At around the time of this study, in non-tsunami-effected Indonesia, private health care was the major source of care (World Bank, 2006). In our study it was found that the usage of public health care was approximately double that of private care, in contrast to the rest of the country at the same time. An area of future research could be to determine if higher perceived satisfaction is correlated with higher actual quality of care in this setting or in a similar post-disaster setting.

Given the utility of mobile clinics and their high accessibility, both in terms of distance from the home and low cost of services and medicine, this may be an area to consider increasing in future similar efforts. This effective source of health care could be augmented and provided more systematically by the relief community in order to reach a greater percentage of the sick children who need health care. Mobile clinics could be used to target those populations that seek less care for their sick children, including displaced populations, and those children whose mother or both parents had died (Rassekh & Santosham, 2014).

With 78.3% of all respondents having utilized formal health care as their first line of care for their sick child (Rassekh & Santosham, 2014), it seems that the health system responded well to the needs of sick children after the tsunami. Overall, the public healthcare system, in cooperation with the relief community's efforts and the local private care system, managed to provide accessible health care in terms of distance, cost, and medicine availability to both displaced and non-displaced populations residing in the study area.
